# Supplementary material for: Whole genome sequencing of SARS-CoV2 strains circulating in Iran during five waves of pandemic
Source: PLoS One. 2022 May 2;17(5):e0267847. doi: 10.1371/journal.pone.0267847 (PMC9060343; doi:10.1371/journal.pone.0267847)
Supplement: Attachment — Submitted filename: Response to Reviewers.docx [file pone.0267847.s001.docx]

**Dear Dear Editor-in-Chief of PLOS ONE,**

Thank you very much for the review of our manuscript entitled: “Whole genome

sequencing of SARS-CoV2 strains circulating in Iran during five waves of

pandemic”.

We sincerely appreciate all valuable comments and suggestions, which helped

us to improve the quality of the article. Our responses to the Reviewers’ comments

are described below in a point-to-point manner. Appropriated changes, suggested

by the Reviewers, have been introduced to the manuscript (highlighted within the

document). The language is improved.

**Dear Reviewer 1:**

The changes were highlighted in yellow.

- Please mention to the COVID-19 in the introduction section.

The sentence was added to the beginning of the Introduction.

- Please mention to the different known clades of SARS-CoV-2 in the introduction section.

Was added to the last paragraph of Introduction.

- In the figure legend please replace “constricted” by “constructed”

Done.

- The number of viruses in the phylogenetic tree is 53.

Phylogenetic tree was corrected.

- Since the purpose of the manuscript was finding information about the viral lineages, variants of interests and variants of concern, it would be suggested to describe them briefly.

Was described at the end of Introduction.

- Please add the following reference “Usage of peptidases by SARS-CoV-2 and several human coronaviruses as receptors: A mysterious story” in the line 366.

Was added.

**Dear Reviewer 2:**

The changes were underlined.

- The Introduction mostly explains the virus proteins and structure, while it might be preferred that first the Introduction part talks also about why this virus is important why this study was designed.

It was added to the end of Introduction.

- It is suggested that the mutations in different proteins (non-structural, structural and accessory) are entered and organized in a table, so the reader can easily see and follow different mutations in different proteins during different waves of the pandemic in Iran.

Mutations of nonstructural genes illustrated in the figure.

- Line 486: … which the results were compatible with … -- needs to be revised to: … of which the results were compatible with …

Corrected.

**Dear Reviewer 3:**

The changes were highlighted in green.

This is a thorough analysis of the genome sequences of SARS-CoV-2 viruses in circulation in Iran over five waves of the pandemic. The full genome sequence of five to twenty viruses (10,10, 9, 5 and 20) from each wave were analyzed by next generation sequencing and the results analyzed to report here on what variation has been seen.

- The data quality has not been described fully: for example, how complete each genome sequence turned out to be and what proportion of undetermined nucleotides were in the genome sequence assembly. This would provide some kind of quality assessment of the gene sequence data. The gene sequence data have been shared in the EpiCov database of GISAID, but I have not checked the sequences fully.

Added to the materials and methods section.

- The catalogue of changes seen and described in the results is comprehensive, but going through the results for each virus polypeptide is demanding for the reader and I wondered if the authors could come up with some kind of graphical display to supplement the description of what was seen and elaborated on in the text. I think for most virus polypeptides this could be relatively easily done, but, like in spike (for which there is a dedicated table), the variation in NSP3 seems quite extensive. Nevertheless, I think a graphical representation would enhance the message of the manuscript greatly.

Mutations of nonstructural genes illustrated in the figure.

- In the discussion the authors summarize a lot of literature on the likely effects of amino acid substitutions. My feeling is that the authors should differentiate between results and conclusions made by experiment from those that have been generated by modelling. Moreover, there is frequent use of phrases like ‘viral oligomerisation interface’ (e.g. lines 136, 140, 141, 147, 150, 151 etc.). Here I was not clear in many cases what the evidence for this conclusion was. I might have missed the references to many of these. In addition, it was not what was meant precisely by a ‘viral oligomerisation interface’. I was not sure of this meant oligomerisation of the relevant polypeptide or something else.

That is really important point. There is not any reference about the role of some proteins as viral oligomerization interfaces, we added this, just extracted from GISAID mutation analysis, then we deleted them.

- There are also two places in which antibody recognition sites are described for the NSP7 and NSP8 (lines 175 and 179). Do the authors know if this recognition is by post-infection serum or something else? Also, references should be given for this. NSP7 was also stated to undergo antigenic drift. I wonder if this is better defined as antigenic change, or change in a T-cell epitope. This is also referred to on line 314.

It is another important point which is written just according to GISAID mutation analysis and there is not publication or in vitro studies, then we removed those from the article.

- The phylogenetic tree shown in the figure uses the GISAID clade nomenclature. I wonder if it is possible to correlate these clades and subclades with the PANGO lineages.

We could change the clades to PANGO in the tree, but because we used mostly

clades in the text, then we prefer to put clades, if it is necessary we could

change them.

- I think it might also be useful to include a graph of the five waves of COVID-19 in Iran and indicate when the samples were taken on the graph.

It is useful to include a graph, but because of the article length and number of tables and figures, we just wrote the date of each wave and the number of assessed samples in each wave.

Minor points.

- In many cases the authors refer to mutations in the virus polypeptides. I think, strictly speaking, mutations occur in genes that encode mutant proteins that have amino acid substitutions. I would recommend that this is considered by the authors.

All were corrected.

- There are a number of places where the English is not correct – the indefinite and the definite articles are missing in some number of places. This should be corrected by asking for additional help from colleagues.

Reviewed and corrected as much as possible.

Some other points include:

- Generally, use family names only when referring to work done by others.

All were corrected.

- Also, the word ‘data’ has been used both as a singular term (incorrectly) and as a plural (correctly). There should be consistency.

Corrected.

I would suggest that when referring to a series of amino acid substitutions the order of these substitutions is always from the N-terminal to C-terminal of the polypeptide.

Corrected

- Line 62, define what a ‘primase’ is.

Added.

- Line 69. I suggest saying that Spike ‘mediates’ attachment and entry rather than is ‘important for’ attachment and entry.

Corrected.

- Line 88. I think ‘designated’ is not the right word here – perhaps ‘selected’.

Corrected.

- Line 98. I suggest ‘Library construction was done by using…’

Corrected.

- Line 99. Explain what ‘captured’ means in this context.

The sentence was changed to clear explanation.

- Line 236. Define in which wave hCoV-19/Iran/Gilan/NICS1-58/2020 was isolated.

Cleared.

- Line 295. The word ‘third’ should be ‘thirds’.

Corrected.

- Line 305. “Recommended’ seems to be the wrong word here.

Corrected.

- Line 346. Add the words ‘change in’ ahead of ‘incubation period’.

Added.

- Line 366. I think this should be Receptor Binding Domain (not Receptor Binding Motif).

Corrected.

- Lines 455 to 456. I am not clear what intraviral connections refers to. Lines 453 to 457 might be better composed.

Corrected.

- Line 481. The authors comment on the ‘rate’ of mutation. However, mutations rates have not been addressed in this work. This needs re-wording to reflect more accurately what has been observed in this study.

It corrected. We mean the number of mutations.
