# Supplementary material for: Whole genome sequencing of SARS-CoV2 strains circulating in Iran during five waves of pandemic
Source: PLoS One. 2022 May 2;17(5):e0267847. doi: 10.1371/journal.pone.0267847 (PMC9060343; doi:10.1371/journal.pone.0267847)
Supplement: Attachment — Submitted filename: Response to Reviewers2.docx [file pone.0267847.s003.docx]

**Dear Dear Editor-in-Chief of PLOS ONE,**

Thank you very much for the review of our manuscript entitled: “Whole genome

sequencing of SARS-CoV2 strains circulating in Iran during five waves of

pandemic”.

We sincerely appreciate all valuable comments and suggestions, especially dear reviewer 3, which helped us to improve the quality of the article. Our responses to the Reviewers’ comments are described below in a point-to-point manner. Appropriated changes, suggested by the Reviewers, have been introduced to the manuscript. The language is improved by all corrections made by reviewer 3.

**Reviewer #3**: The manuscript by Yavarian et al. has addressed many of the points I raised in my report. However, I think that there are still various sections of the manuscript where improvements to the flow or meaning can be made. I will attach a scan of a handwritten marked-up document for the authors to consider. However, there are places that the authors still need to address.

The main places where the authors need to further modify the text are as follows. (Minor changes suggested are in the marked-up copy).

**Lines 108 to 110**. The term ‘mutations’ remain in this section to be used for amino acid substitutions. The same is also seen in lines 113 and 116. I suggest the authors re-check for the appropriate usage. I have made some suggestions in the marked-up scanned copy.

They all corrected.

**Line 140**. Introduce the concept here of amino acid deletions e.g. using the phrase ‘a deletion of G66 (G66del) and S76 (S67del)…’ See also lines 162 to 164, and line 240.

They all corrected.

**Lines 144 and 145**. Introduce the concept of stop codons in NS7b and NS8 - and the effect of the stop codons on what can be expected to be translated.

We added that stop codons lead to truncated proteins.

**Line 171** is not clear what the authors are trying to say here. Perhaps it is that amino acid substitutions in NS3 deserve greater study in vitro.

It was re-written.

**Lines 195 to 197**. I have made suggestions for changes here in the marked copy.

Correction was made.

**Line 211**. It was not clear what the authors meant by ‘missense mutations’.

We wrote change in amino acid.

**Line 243**. A reference is needed.

Added.

**Line 271**. It was unclear what therapeutic effects were envisaged here.

It was not appropriate sentence, we removed it.

**Line 276**. Here the authors are presumably referring to amino acid substitutions in the nucleoprotein, not in the gene.

Corrected.

**Line 277**. A reference is needed.

Added.

**Line 287**. A reference is needed.

Added.

**Lines 289 to 291**. It is not clear what the authors are saying that termination of the NS8 gene somehow affects spike affinity for the receptor. The authors should elaborate on this.

We re-wrote it.
